# Physician-assisted suicide in Italy: where do we stand and where do we want to go?

**DOI:** 10.3389/fpsyt.2025.1606036

**Published:** 2025-07-09

**Authors:** Emanuela Turillazzi, Donato Morena, Naomi Iacoponi, Vittorio Fineschi

**Affiliations:** ^1^ Department of Surgical, Medical and Molecular Pathology and Critical Care Medicine, University of Pisa, Pisa, Italy; ^2^ Department of Anatomical, Histological, Forensic and Orthopedic Science, Sapienza University of Rome, Rome, Italy

**Keywords:** physician assisted suicide, physician assisted suicide law, end of life, life sustaining treatments, life-ending decisions, terminal care

## Abstract

Physician-assisted suicide (PAS) remains a subject of global debate and ethical controversy, alongside other end-of-life issues such as euthanasia, palliative care, access to health and social care services, and, importantly, patient autonomy. Within this context, PAS is defined as the practice in which a physician, at the explicit request of a competent patient, prescribes a lethal medication that the patient may self-administer to end their own life. This definition clearly distinguishes PAS from euthanasia, wherein the physician directly administers the life-ending drug. Despite ongoing efforts to establish a unified definition, significant variability remains across jurisdictions with regard to eligibility criteria, procedural safeguards, and the overarching legal and ethical frameworks governing PAS. In Italy, there is currently no effective legislation regulating euthanasia or PAS, which remain practices punishable under Articles 579 (homicide of a consenting person) and 580 (instigation or assistance in suicide) of the Italian Criminal Code. The Tuscany Region has prepared a regulatory attempt at the regional level, which has, however, limited itself to proposing an operational protocol that distinguishes the operational responsibilities that the PAS procedure applicant will face. However, following several relevant cases that profoundly influenced the Italian debate, significant attention has been directed toward end-of-life issues.

## 1 Introduction

Physician-assisted suicide (PAS) remains a subject of global debate and ethical controversy, alongside other end-of-life (EoL) issues such as euthanasia, palliative care, access to health and social care services, and, importantly, patient autonomy. Within this context, PAS is defined as the practice in which a physician, at the explicit request of a competent patient, prescribes a lethal medication that the patient may self-administer to end their own life (1, 2). This definition clearly distinguishes PAS from euthanasia, wherein the physician directly administers the life-ending intervention. Despite ongoing efforts to establish a unified definition, significant variability remains across jurisdictions with regard to eligibility criteria, procedural safeguards, and the overarching legal and ethical frameworks governing PAS (3).

In Italy, there is currently no effective legislation regulating euthanasia or PAS, which remain practices punishable under Articles 579 (homicide of a consenting person) and 580 (instigation or assistance in suicide) of the Italian Criminal Code (4). The Tuscany Region has prepared a regulatory attempt at the regional level, which has, however, limited itself to proposing an operational protocol that distinguishes the operational responsibilities that the PAS procedure applicant will face (5).

However, following several relevant cases that profoundly influenced the Italian debate (6), significant attention has been directed toward EoL issues.

A particularly significant case is that of Fabiano Antoniani, widely known as “DJ Fabo”, who sustained brain injuries in a traffic accident in June 2014. Although his cognitive functions remained intact, the injuries resulted in tetraplegia and bilateral cortical blindness (7, 8). These injuries rendered his breathing non-autonomous, requiring, although not continuously, the assistance of a ventilator and periodic suction of mucus; his nutrition required artificial feeding, and evacuation was also dependent on assistance (9). Additionally, he suffered from recurrent painful contractions and spasms, which were resistant to medication. Unable to access assisted suicide in Italy, he first contacted Dignitas, a Swiss non-profit organization providing legal assistance for voluntary death, and then the “Luca Coscioni Association”. In February 2017, he was assisted by a member of the latter association in traveling to Switzerland, where he ended his life. Upon his return to Italy, the person who had helped him was investigated for incitement and aiding suicide. Following a judicial process to resolve certain points of conflict with specific articles of the Italian Constitution and the European Convention on Human Rights (ECHR), the Constitutional Court was tasked with evaluating the case. The first step in this process was the ruling of Ordinance No. 207 on February 1, 2018 (10). The Court clarified that states can intervene in the exercise of the aforementioned right to self-determination, including criminalizing assistance in suicide, provided that such intervention is necessary and proportional to protect the rights and freedoms of others, including the position of vulnerable individuals, who are generally considered to be those with suicidal intentions. The Court specified that in certain situations, “third-party assistance in ending one’s life may present itself to the patient as the only way to avoid, in accordance with their own concept of personal dignity, an artificial life-sustaining process they no longer wish to endure and that they have the right to refuse”. This right emanates from Article 32 §2 of the Constitution and has been specifically applied in the formulation of Law No. 219/2017 (law on informed consent and advance directives). According to this law, the Court noted, the patient, when adequately informed about available alternatives and supported psychologically, has the right to refuse life-sustaining treatments (LSTs), including ventilation, artificial nutrition, and hydration, and to be accompanied to death with continuous deep palliative sedation associated with pain therapy. Specifically, the cases where Article 580 of the Criminal Code may not apply are those conditions in which the individual seeking to end their life is (a) suffering from an irreversible disease, (b) experiencing intolerable physical or psychological suffering, (c) being kept alive through life-sustaining treatments (specifically “ventilation, artificial hydration, or feeding”), and (d) capable of making free and informed decisions. Furthermore, under the principle of “deferred constitutionality”, the Court referred the matter to Parliament, requesting a legislative initiative that considers legal evolution. In September 2019, the Court would assess the potential enactment of a regulating law that addresses the identified needs for protection. Approximately one year after the 2018 ordinance, the Constitutional Court, recognizing the legislator’s inaction in providing detailed regulation on the matter, affirmed that it could no longer abstain from ruling in order to address the constitutional deficit previously identified. With Judgment No. 242 of September 25, 2019, the Court reaffirmed the constitutional illegitimacy of Article 580 of the Criminal Code, limited to cases where individuals assisting in the execution of a suicide, freely and autonomously decided, in accordance with Articles 1 and 2 of Law No. 219/2017, could meet the four criteria outlined in the 2018 Ordinance. Further requirements were thus established for requesting access to assisted dying, with the Court deeming it necessary that:

- all of the aforementioned conditions be verified within a medical context;
- the individual’s will be expressed clearly and unequivocally, to the extent permitted by their condition;
- the patient be adequately informed both about their medical condition and the available alternative options, particularly with regard to the possibility of receiving palliative care and, if applicable, continuous deep sedation.

The Court identified public facilities within the National Health Service (NHS) as the appropriate entities responsible for verifying both the conditions legitimizing PAS and the corresponding procedures. To further safeguard particularly vulnerable individuals, the involvement of a third-party collegial body was mandated. In the absence of specific legislative measures, this role was provisionally assigned to the territorially competent Ethics Committees (ECs). These institutional bodies serve as consultative and reference entities for ethical issues that may arise in healthcare practice. They are endowed with only advisory functions, aimed at ensuring the protection of a person’s rights and values in relation to any clinical trials involving humans.

Regarding the issue of conscientious objection by healthcare personnel, the Court emphasized that no obligation exists for physicians to participate in assisted dying. The decision to provide such assistance remains a matter of individual conscience.

Following the 2019 pronouncement of the Constitutional Court, several requests for PAS have been made in Italy, some of which led to access to PAS, while others resulted in further judicial disputes, culminating in a recent judgment from the Constitutional Court, which is still addressing the issue of PAS (11).

The purpose of this contribution is to analyze the Italian PAS cases following the 2019 Constitutional Court pronouncement, which, as discussed below, have once again renewed the Italian debate on the topic and led to a further judgment from the Supreme Court.

## 2 PAS: an assessment of options and implications in Italy

### 2.1 Background

Since the regulatory framework established by the Italian Constitutional Court’s 2019 judgment created the possibility for medical aid in EoL in the specific cases outlined and according to the prescribed procedure for PAS decriminalization, requests and applications for PAS are increasing.

As showed in the 57th Report on the social situation of the Italian society in 2023, drawn up by CENSIS (12), “among 74% of Italians say they are in favor of euthanasia, with percentages across the social body, reaching 82.2% among young people and to 79.2% among graduates”. However, four years after the publication of the 2019 Constitutional Court judgment, numerous obstacles still persist, preventing interested individuals from accessing PAS. Amid prolonged delays in legislative regulation, several judicial decisions have been issued, underscoring the challenges of addressing such a complex issue without regulatory frameworks and relying solely on Constitutional Court interventions.

In the absence of official data on PAS, according to the press release published on the Luca Coscioni Association’s website on 1 April 2025, to date 51 requests have been received in various regions with varying outcomes including approvals, denials and ongoing procedures (13).

In the continued absence of legislative intervention by the state, the NHS has failed to ensure timely and adequate procedures as mandated by Constitutional Court Judgment No. 242/2019 (assessment of the patient’s medical eligibility; identification of the appropriate lethal drug and its method of self-administration; verification of the patient’s clear and unequivocal intent; and confirmation that the patient has been fully informed about their condition and possible alternatives, such as access to palliative care and, where appropriate, continuous deep sedation). As a result, since 2019, numerous requests for the initiation of the PAS procedure have been brought before the courts due to delays by health authorities or uncertainties concerning the fulfillment of the criteria established by the Constitutional Court.

**Figure 1:**
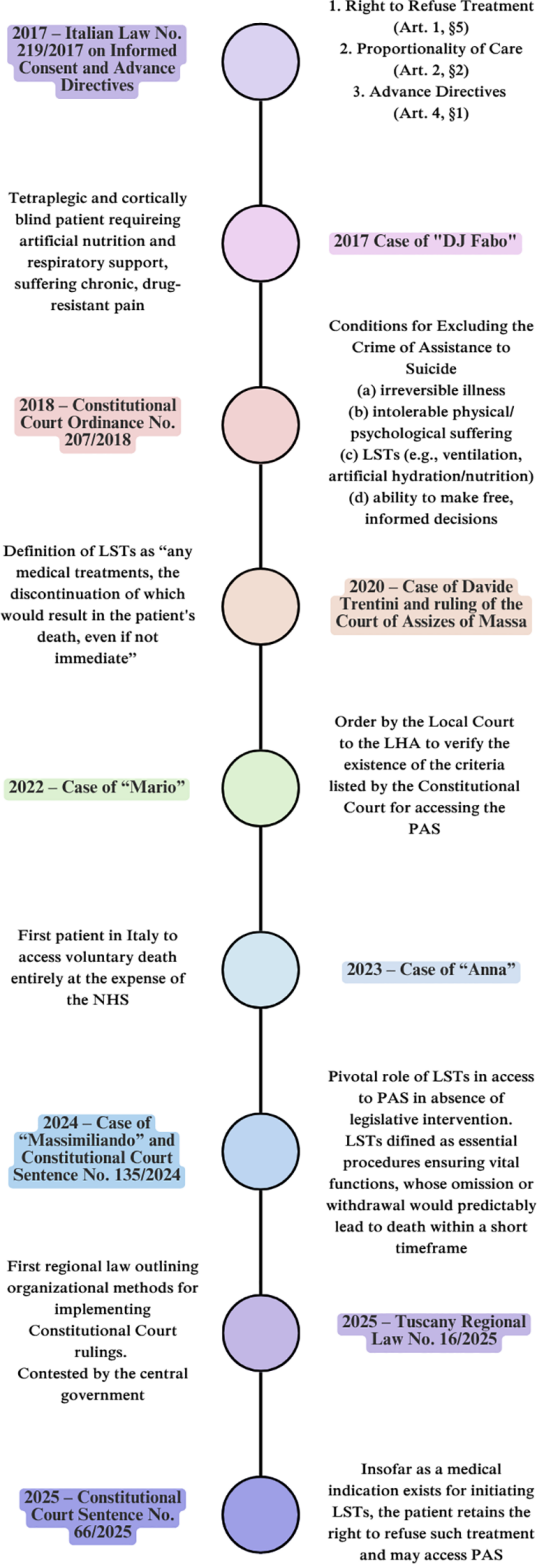
Timeline of PAS legal evolution in Italy. LHA, Local Health Authority; LSTs, Life-Sustaining Treatments; NHS, National Health Service; PAS, Physician-Assisted Suicide.

This has led to a fragmented and inconsistent national landscape. Even in the presence of the conditions stipulated by Judgment No. 242/2019, some courts have rejected the existence of an obligation on the part of local health authorities to provide assisted suicide services, including the administration of the necessary drugs (14), while others have affirmed such an obligation (15). This divergence has resulted in serious disparities in the treatment of patients in comparable clinical and legal circumstances. In this context—marked by significant challenges in the implementation of the 2019 Constitutional Court Judgment, primarily due to the absence of national legislation—a series of legal cases and a limited number of regional regulatory initiatives (**Figure 1**) have reignited debate, particularly concerning the criterion of dependence on LSTs.

### 2.2 Leading cases

#### 2.2.1 The case of Mario

The case of “Mario” (a fictitious name chosen by the patient himself, F.C.), the first Italian patient to access PAS, is emblematic. In 2010, Mario became tetraplegic due to a severe spinal injury sustained in a road accident, leaving him dependent on intensive LSTs. After enduring years of unbearable suffering, he made the decision in 2020 to end his life through PAS. The process leading to his death on June 16, 2022, began with a request to the Health Authority of the Marche Region to evaluate whether he met the criteria established by the Constitutional Court for PAS access. Initially, the regional Health Authority denied his request. In response, Mario filed an appeal with the regional Court of Ancona, which initially rejected his petition to compel the Health Authority to prescribe the drug required for a “quick, effective, and non-painful” death (16). Undeterred, Mario submitted a second appeal. This appeal overturned the previous ruling and directed the Marche Health Authority to evaluate his eligibility for PAS, as defined by the Constitutional Court, and to verify the appropriateness and effectiveness of the proposed method and drug for ensuring a rapid, painless, and dignified death. The evaluation required consultation with the EC of the relevant jurisdiction. On November 9, 2021, the EC of the Marche region issued a favorable opinion, which was followed by a clarification on November 23, 2021, stating that the final decision on Mario’s right to access PAS rested with the Court of Ancona.

Subsequently, in January 2022, the Marche Health Authority identified an appropriate drug: sodium thiopental, a rapid-onset short-acting barbiturate general anesthetic, determined to be capable of ensuring a rapid and painless death at a dosage of 3–5 grams for an adult weighing 70 kg. The specified method of administration was self-administered intravenous infusion. Finally, on June 16, 2022, two years after his initial request, Mario was able to access PAS.

This case underlies statements related to the fundamental requirements of Constitutional Court Judgment No. 242/2019, which references Law 219/2017, specifically concerning:

- the requirement that the will of the individual must have been expressed clearly and unequivocally, insofar as permitted by their condition; and
- the need for this will to be assessed in light of the fact that the patient must have been adequately informed both about their condition and about possible alternative options, particularly with regard to access to palliative care and to deep continuous palliative sedation.

#### 2.2.2 The case of Anna

In a different sense the decision of the court of Trieste (4 July 2023) in the case of “Anna” (fictitious name), a woman approximately 55 years old suffering from progressive multiple sclerosis, prolonged delays in verifying her physical condition led to judicial intervention (17). The competent judge ordered the local health authority to pay a penalty for each day of delay in fulfilling its obligations. Only after this ruling did the health authority finally provide the interested party with the necessary medical assistance, including the lethal drug and the equipment required for its self-administration, with all costs covered by the NHS. “Anna” is thus the first patient in Italy to access voluntary death entirely at the expense of the NHS.

It is a matter of fact that the structural differences within the Italian healthcare system—where regional authorities hold near-exclusive responsibility for the organization and delivery of healthcare services—combined with the persistent absence of national guidelines and standardized protocols, have contributed to significant delays in the verification of patients’ medical conditions by local health authorities and the territorially competent ECs. Furthermore, procedural uncertainties—such as those related to the type, quality, and method of administration of the lethal drug—have, in some cases, necessitated judicial intervention, often resulting in inconsistent and divergent rulings.

Undoubtedly, the current procedures for accessing PAS reveal significant limitations, foremost among them being the absence of regional legislation that—pending the adoption of a national law—would ensure clear timelines for the verification process. To date, Tuscany remains the only region to have enacted such a law. On 14 March 2025, Regional Law No. 16/2025 was approved, titled “Organizational Methods for the Implementation of Constitutional Court Judgments No. 242/2019 and No. 135/2024”, aimed at ensuring a regulated and uniform process for access to PAS (5).

The law establishes that anyone requesting an assessment of their health conditions to access PAS must receive a response within a maximum of thirty days. More specifically, the first phase is the verification of the requirements indicated by the aforementioned judgments of the Constitutional Court by a Commission composed of various figures such as anesthetist, psychiatrist, psychologist, medical examiner, nurse, and integrated, from time to time, by a doctor specialized in the pathology from which the person requesting access to PAS is affected. The Commission is required to request the opinion of the EC on the ethical aspects of the case in question. In the event of a positive outcome and confirmation of the choice, assistance must be provided within a further seven days. However, the Italian government has challenged the Tuscan regional law, arguing that the regulation of such matters falls within the exclusive competence of the national legislature.

#### 2.2.3 The case of Davide Trentini

Davide Trentini (18), suffering from multiple sclerosis, requested PAS and, therefore, was accompanied by Marco Cappato and Mina Welby to Switzerland, where he was administered an intravenous injection of the lethal drug, using an injection mechanism that he activated himself, leading to death within minutes. Upon returning to Italy, Marco Cappato and Mina Welby were charged with incitement to and aiding in suicide. The Assize Court of Massa Carrara acquitted the accused of the charges of incitement and assistance to suicide. Simultaneously, the judges addressed the issue of dependence on LSTs (19).

The most controversial aspect of the Mr. Trentini case, and the reason why the ruling of the Court of Assizes in Massa was particularly anticipated, was the question of whether the requirement of “dependence on life-sustaining treatments”, as defined by the Constitutional Court, could be considered a constitutive element of the cause for non-punishment under Article 580 of the Criminal Code. Indeed, Mr. Trentini was not connected to machines for feeding, breathing, or administering drugs. He had been suffering from multiple sclerosis since 1993. Over time, the disease became progressively non-remitting: it advanced slowly but inevitably, rendering recovery impossible and leading to increasingly painful conditions. Mr. Trentini required assistance to get out of bed or take a shower; his gait became progressively ataxic and paraparetic, necessitating the use of a walker. He often fell and experienced pain that became so intense it was unbearable.

During the court trial, the expert witness testified that, in the final years of his life, Mr. Trentini could indeed be said to be dependent on two forms of life support: one pharmacological and the other mechanical.

Firstly, Mr. Trentini received targeted pharmacological therapy primarily to manage pain, which had reached intolerable levels. An increase in the dosage, repeatedly requested by the patient, would have resulted in a drug overdose, while a reduction in dosage would have led not only to unbearable pain but also to worsening respiratory failure, thus accelerating the progression of the disease and leading to death. Secondly, Mr. Trentini had lost autonomy for bowel movements due to the paralysis of his intestinal muscles, requiring manual evacuations, initially performed by healthcare workers and later by his mother. Without this intervention, the inevitable consequence would have been intestinal perforation, which would have resulted in his death.

The Court of Massa concluded that the pathology from which Mr. Trentini suffered was irreversible and caused both physical and psychological suffering. As demonstrated by his firm intention to end his life, he found this suffering absolutely intolerable. It was also determined that, despite his suffering, Mr. Trentini remained capable of making free and informed decisions. Finally, the judges accepted the expert’s testimony regarding the dual (pharmacological and mechanical) dependence that characterized Mr. Trentini’s condition. This dependence satisfied the final criterion established by the Constitutional Court for the legal exemption from liability for assisted suicide. According to the judges, Mr. Trentini was not autonomous in his basic vital needs. His situation was akin to that of individuals who, to continue living, “depend on others” to assist them with moving, eating, and using the bathroom. If a person depends on others (whether people or devices) to meet their vital needs, the requirement defined by the Constitutional Court can be considered fulfilled. However, the issue of LSTs continued to evolve in Italy.

#### 2.2.4 The case of Massimiliano

In January 2024, the Court of Florence raised the question of the constitutional legitimacy of Article 580 of the Criminal Code, “as modified by Judgment No. 242 of 2019 by the Constitutional Court”, particularly the provision requiring the legal exemption from liability of individuals who facilitate the suicide of others, contingent upon the circumstance that the aid is provided to a person “kept alive by life-support treatments”.

Doubts were raised regarding the constitutional legitimacy of the requirement in question, specifically with reference to Article 3 of the Constitution (on the grounds that it results in an unreasonable disparity of treatment between substantially identical situations); Articles 2, 13, and 32 §2 (as the requirement would entail an undue restriction on the patient’s freedom of self-determination in therapeutic choices); and Article 117 of the Constitution in connection with Articles 8 and 14 of the ECHR, insofar as it constitutes an interference with the right to respect for private and family life that is not justified by the protection of the right to life (20). The case involved three members of the “Luca Coscioni Association”, who are under investigation for facilitating the suicide of a man, “Massimiliano”, who suffered from multiple sclerosis, by accompanying him to Switzerland for access to PAS. The patient, after the worsening of his condition and a decline in his health, began contemplating ending his life in 2021. By 2022, his intention to die had strengthened, leading him to contact a Swiss clinic with the mediation of Marco Cappato, the legal representative of the association. Mr. Cappato provided financial support, covering the costs of the procedure and the transportation of the patient to Switzerland. On December 8, 2022, after confirming his wish to end his life, Massimiliano independently ingested the lethal drug with the arm he could still control, dying a few minutes later.

As in the Mr. Trentini case, once back in Italy, the members of the “Luca Coscioni Association” were charged with incitement to and aiding in suicide. The Judge of Florence ruled that the actions did not fall within the non-punishment provision introduced by Article 580 of the Criminal Code following Judgment No. 242/2019 of the Constitutional Court, as “the requirement of ‘dependence on life-support treatments’ was not met”. In this case, the patient was not kept alive by life-support devices, nor did the advanced stage of his illness require such treatments. Consequently, the judge raised the question of the constitutional legitimacy of the requirement set forth in Judgment No. 242/2019 (21) considering whether the condition of being “kept alive by life-support treatments” could potentially conflict with the principles of the Italian Constitution.

This led to the Constitutional Court’s Judgment, in which the criteria for the recognition of LSTs were also reformulated (22), as discussed below.

## 3 The key question of life-sustaining treatments: the Constitutional Court’s response and actionable recommendations

The central issue, therefore, revolves around dependence on LSTs, a concept that the Constitutional Court did not explicitly define in 2019. Instead, the Court provided examples, such as ventilation, hydration, or artificial nutrition. This lack of definition has led to significant interpretive debates. On one hand, some argue that the requirement should not be limited to mere “dependence on machinery”, as it can encompass a broad range of situations where LST is provided through pharmacological therapies or assistance from medical or paramedical personnel. In other words, any treatment whose interruption would result in the patient’s death—whether immediately or over time—would constitute a LST.

A broader interpretation suggests that the requirement would also apply when a patient relies on third-party assistance for essential activities such as eating, moving, or using the bathroom. The underlying principle is that a situation where an individual needs help from others (family members or others without specific medical training) to perform vital functions shares similarities with cases where health interventions (mechanical, pharmacological, or assistive) are required to sustain life.

Dependence on LSTs may also arise when a patient, despite having intact bodily functions, progressively becomes unable to perform daily physiological activities due to limb immobilization and requires increasing assistance from others.

These varied interpretations, coupled with the absence of a clear definition of LSTs, led the Court of Florence to request that the Constitutional Court clarify the concept of “LST”.

The Constitutional Court first reaffirmed that the requirement of the patient’s dependence on LTSs has ‘in the absence of legislative intervention, a pivotal role within the rationale of the solution adopted in Ordinance No. 207 of 2018, later reiterated in Judgment No. 242 of 2019’ (22).

However, the Constitutional Court avoided providing a strict definition or limiting the concept of LSTs. Instead, the Court expanded the definition significantly. Thus, regardless of the invasiveness or technical nature of the treatment, any procedure deemed necessary to ensure the performance of vital functions—such that its omission or interruption would predictably lead to the patient’s death within a short time—must be considered as LST for the purposes of applying the principles established in Judgment No. 242/2019. This broader interpretation includes procedures like manual evacuation, catheter insertion, or bronchial mucus aspiration—typically performed by healthcare personnel but which can also be carried out by family members or caregivers, provided that discontinuing these procedures would predictably result in the patient’s death within a short time.

The Constitutional Court reaffirmed this position in its recent Judgment No. 66/2025 (23).The judgment refers to two cases involving individuals who were not being kept alive by LSTs, but such treatments had been prescribed by physicians and subsequently refused by the patients:

- the case of E.A., a patient diagnosed with metastatic small cell lung cancer who, due chemotherapy ineffectiveness, decided to stop treatment and refused the life-sustaining measures proposed to address worsening respiratory and kidney functions. The patient ended their life at a clinic in Switzerland after paying approximately ten thousand euros;
- the case of R.N., a patient suffering from atypical, rapidly progressing Parkinson’s disease, which had led to a complete loss of autonomy in daily activities and the ability to swallow. A percutaneous endoscopic gastrostomy was proposed, but the patient declined the intervention. Cognitive abilities, however, remained intact. The patient ended their life at a clinic in Switzerland.

The Judgment issued by the Constitutional Court thus reaffirmed that, insofar as there exists a medical indication for the initiation of an LST—as specified in Judgment No. 135/2024—the patient retains the right to refuse such treatment and, provided that the other substantive and procedural requirements set out in Judgment No. 242/2019 are met, may access PAS.

## 4 Discussion

Despite recent judgments by the Constitutional Court, the debate regarding the requirement of dependence on LSTs as a condition for accessing PAS remains unresolved.

In this context, the issue of LSTs continues to be pressing. Recent developments have prompted a deeper examination of the matter in Italy at multiple levels. For instance, during a hearing before the Justice and Health Committees on PAS-related legislation, a representative from the Ethics Committee of the Italian Society of Anesthesia, Analgesia, Resuscitation and Intensive Care emphasized the importance of primarily considering the subjective experience of burdensomeness (24). When discussing the proportionality of care, two essential factors must be balanced: clinical appropriateness and the burdensomeness experienced by the patient. The healthcare burden, as subjectively perceived by the patient, may exceed their coping capacity, leading to a sense of being overwhelmed. As has been noted, perceived burdensomeness can result from both external and individual factors. Among the latter, the patient’s sense of autonomy is particularly significant and may be compromised by ongoing dependence on care (25).

Furthermore, the subjective dimension is crucial. If a patient perceives themselves as a burden but is not emotionally distressed by this perception, it may not contribute to unbearable suffering. Conversely, when care needs and dependency—irrespective of the nature or invasiveness of treatment—exceed the patient’s capacity to cope, perceived burdensomeness may become a substantial component of intolerable suffering. This experience is inherently subjective and can only be accurately assessed by the patient themselves (3, 26–28), as many medical conditions may lead to severe suffering and functional decline without impairing cognitive ability or decision-making capacity.

This is a judgment that should rightfully belong to the competent patient. If it is ethically and medically acceptable for a conscious patient to refuse or discontinue even LSTs, then it appears equally reasonable for a competent and conscious individual to make similar determinations in the context of a PAS request. We believe that the concepts of life’s value, personal autonomy, and the benefit–burden analysis should be central to our understanding of personhood.

The issue becomes more complex when addressing patients who are suffering but not dependent on LSTs for survival and who have no prospect of recovery. Severe illnesses—such as parkinsonian syndromes, multiple sclerosis, or neuromuscular diseases—often entail a progressive loss of functional autonomy. Our experience in local ECs indicates a growing number of patients in such conditions requesting access to PAS, precisely due to the progressive loss of personal autonomy, particularly with respect to basic functions such as eating, drinking, and elimination.

These individuals frequently anticipate the natural progression of their illnesses by drafting Advance Healthcare Directives (DATs), explicitly refusing future invasive treatments (e.g., percutaneous endoscopic gastrostomy-PEG feeding or mechanical ventilation), as well as any medical intervention required to maintain basic vital needs.

A significant shift occurred with the case of Davide Trentini and the ruling by the Massa Court, which recognized that “any medical treatment whose interruption would result in the patient’s death, even if not immediately, is equivalent to LSTs”.

Nevertheless, a legal and ethical gap persists regarding forms of assistance that, while not strictly medical, are essential for survival—for example, caregiver-provided support with nutrition. In such cases, the absence of clear jurisprudence has led healthcare authorities to deny authorization for PAS. This was the case with Martina Oppelli, a 49-year-old woman with secondary progressive multiple sclerosis (29), whose disease had progressed to cause severe motor impairment, pain, and uncontrollable spasms, leaving her entirely dependent on others for all daily activities. Her dependence on non-medical caregivers resulted in the local health authority’s determination that the criterion of “dependence on life-sustaining treatments” was not met in her case.

Given these critical issues—marked by discretionary elements and potentially discriminatory risks—we contend that the inability to meet basic vital needs should be recognized as a condition independent of whether the assistance required is medical in nature. Furthermore, we argue that dependence on LSTs does not inherently indicate the severity or progression of a disease, nor does it necessarily reflect the patient’s subjective perception of their condition as intolerable or unacceptable.

Finally, we maintain that perceived burdensomeness is central to PAS decision-making. The patient’s own perception of burden can constitute an essential element of the unbearable suffering required for legal access to PAS. Therefore, this perception—regardless of the type of treatment or support involved—should play a central role in determining eligibility for PAS.

The present study is subject to certain limitations, arising from the fact that it is based on a small number of case histories. These case histories are of national importance and have formed the scientific basis for the debate that is still unresolved.

Furthermore, it reflects the doctrinaire state of the art relating to the Italian legal reality, even though the experience gained from the casuistry may be of valuable help in stimulating the trans-national debate, as is happening.

## 5 Conclusion

Integrating discussions of illness, dependency, and suffering more deeply into the Italian debate on PAS would be highly beneficial. Evidence from several European countries indicates that patient suffering is a key criterion in jurisdictions where PAS is permitted. In the Netherlands, persistent and unbearable suffering constitutes a central justification for PAS, including euthanasia. Similarly, in Belgium, continuous suffering that cannot be otherwise alleviated is a fundamental condition. In Luxembourg, aid to EoL —including euthanasia and PAS—is allowed for individuals with a severe and incurable illness who experience “constant, unbearable physical or psychological suffering”.

In Switzerland, PAS is legally available to individuals with serious, incurable, and chronic mental illness (30).

In Spain, PAS and euthanasia are permitted in cases of a “serious, chronic, and disabling condition or a serious and incurable disease, causing unbearable physical or mental suffering”, with the possibility of applying advance directives (3).

Furthermore, the desire to end suffering and unbearable pain has been identified as the primary reason for requests for assistance in suicide, as demonstrated by a recent demographic study conducted in 13 countries (8 of which are in Europe) where euthanasia or assisted suicide is legal (31).

With regard to the debate on the inclusion of eligibility criteria, and the challenges related to defining LSTs, we propose that the presence of three essential conditions—autonomous suicidal intent by a person capable of making free and informed decisions; an irreversible pathology; and unbearable suffering for the patient — should be both necessary and sufficient for the request for PAS in Italy. This should be considered independently of the issue of LSTs, provided that all conditions and procedures are verified by public structures within the NHS, following the opinion of the territorially competent EC.

Finally, as has been emphasized, it is crucial to collect reliable data in order to assess the standards of procedures and diagnostic work-up related to PAS (2). This debate can foster cultural development, enhance social awareness, and support the creation of shared legislative solutions—not only regionally, but also, as recently reaffirmed by the Constitutional Court, within a unified and practically applicable national legal framework (22, 23).

## References

[1] Solari A Ticozzi N Comi G Mancardi G Provinciali L Padovani A. Neurology and physician-assisted suicide: glossary of definitions and terminology. Neurol Sci. (2025) 46:2381–92. doi: 10.1007/s10072-025-08064-3, PMID: 40009146 PMC12084260

[2] Pucci E Ticozzi N Comi G Mancardi G Provinciali L Padovani A. Neurology and physician-assisted suicide: position of the Italian society of neurology. Neurol Sci. (2025) 46:2371–9. doi: 10.1007/s10072-025-08038-5, PMID: 40014225 PMC12084242

[3] Scopetti M Morena D Padovano M Manetti F Di Fazio N Delogu G. Assisted suicide and euthanasia in mental disorders: ethical positions in the debate between proportionality, dignity, and the right to die. Healthcare. (2023) 11:1470. doi: 10.3390/healthcare11101470, PMID: 37239756 PMC10218690

[4] Riva L. The physician-assisted suicide pathway in Italy: ethical assessment and safeguard approaches. Bioethical Inquiry. (2024) 21:185–92. doi: 10.1007/s11673-023-10302-2, PMID: 37831290 PMC11052828

[5] Regional Law of Tuscany, March 14, 2025, No. 16 – Organizational Procedures for the Medicalized Assistance in Suicide. Available online at: https://www.osservatoriofamiglia.it/contenuti/17520306/legge-regionale-toscana-14-marzo-2025-n-16-modalita-organizz.html (Accessed May 27, 2025).

[6] Botti C Vaccari A. End-of-life decision-making and advance care directives in Italy. A report and moral appraisal of recent legal provisions. Bioethics. (2019) 33:842–8. doi: 10.1111/bioe.12615, PMID: 31264246

[7] Delbon P Maghin F Conti A. Medically assisted suicide in Italy: the recent judgment of the Constitutional Court. Clin Ter. (2021) 172:193–6. doi: 10.7417/CT.2021.2312, PMID: 33956035

[8] Turillazzi E Maiese A Frati P Scopetti M Di Paolo M. Physician-patient relationship, assisted suicide and the Italian constitutional court. J Bioeth Inq. (2021) 18:671–81. doi: 10.1007/s11673-021-10136-w, PMID: 34674155

[9] Cupelli C. Il caso Cappato e i nuovi confini di liceità dell’agevolazione al suicidio. Dalla “doppia pronuncia” della Corte costituzionale alla sentenza di assoluzione della Corte di assise di Milano (2020). Available online at: https://art.torvergata.it/handle/2108/251405 (Accessed May 27, 2025).

[10] Licastro A. Trattamenti sanitari, diritto all’autodeterminazione ed etiche di fine vita dopo l’ordinanza n. 207 del 2018 della Corte costituzionale. In: Stato, Chiese e pluralismo confessionale (2019). Available online at: https://riviste.unimi.it/index.php/statoechiese/article/view/11549.

[11] Italian Constitutional Court Sentence No. 135/2024 (2024). Available online at: https://www.cortecostituzionale.it/actionSchedaPronuncia.do?param_ecli=ECLI:IT:COST:2024:135 (Accessed May 27, 2025).

[12] CENSIS. 57th Report on the Social Situation of the Country (2023). Available online at: https://www.censis.it/rapporto-annuale/57%C2%B0-rapporto-sulla-situazione-sociale-del-paese2023 (Accessed May 27, 2025).

[13] Associazione Luca Coscioni. Almeno 51 le richieste accertate, ma troppe Regioni restano in silenzio (2025). Available online at: https://www.associazionelucacoscioni.it/notizie/comunicati/abbiamo-promosso-un-accesso-agli-atti-sul-suicidio-assistito-almeno-51-le-richieste-accertate-ma-troppe-regioni-restano-in-silenzio (Accessed May 27, 2025).

[14] Associazione Luca Coscioni. Il caso di “Mario” che chiede la morte assistita in Italia e porta in tribunale la Asl punto per punto (2021). Available online at: https://www.associazionelucacoscioni.it/il-caso-di-mario-che-chiede-la-morte-assistita-in-italia-e-porta-in-tribunale-la-asl-punto-per-punto (Accessed May 27, 2025).

[15] Associazione Luca Coscioni. Il caso di “Anna” (2023). Available online at: https://www.associazionelucacoscioni.it/il-caso-di-anna (Accessed May 27, 2025).

[16] Associazione Luca Coscioni. Mario avrebbe diritto al suicidio assistito ma il Tribunale disconosce la sentenza della Consulta (2021). Available online at: https://www.associazionelucacoscioni.it/notizie/comunicati/mario-avrebbe-diritto-al-suicidio-assistito-ma-il-tribunale-disconosce-la-sentenza-della-consulta (Accessed May 27, 2025).

[17] Associazione Luca Coscioni. Il Tribunale di Trieste condanna ASUGI a verificare il diritto di Anna ad ottenere l’aiuto a morire entro 30 giorni (2023). Available online at: https://www.associazionelucacoscioni.it/notizie/comunicati/137738 (Accessed May 27, 2025).

[18] Associazione Luca Coscioni. La storia di Davide Trentini e il processo a Mina Welby e Marco Cappato (2020). Available online at: https://www.associazionelucacoscioni.it/notizie/blog/la-storia-davide-trentini-processo-mina-welby-marco-cappato (Accessed May 27, 2025).

[19] Court of Assizes of Massa, Judgment No. 1 of 27 July 2020 (2020). Available online at: https://www.biodiritto.org/ocmultibinary/download/3939/46390/4/7ac8ce6da583d7be605b7ffc6bd7772f/file/Sentenza-Massa.pdf (Accessed May 27, 2025).

[20] Court of Florence, Order of 17 January 2024 (2024). Available online at: https://www.biodiritto.org/ocmultibinary/download/4529/52920/1/503e324eee6388e11cec61425ebc35b0/file/ordinanza-gip-firenze.pdf (Accessed May 27, 2025).

[21] Italian Constitutional Court, Judgment No. 242 of 2019 (2019). Available online at: https://www.cortecostituzionale.it/actionSchedaPronuncia.do?param_ecli=ECLI:IT:COST:2019:242 (Accessed May 27, 2025).

[22] Italian Constitutional Court, Judgment No. 135 of 2024 (2024). Available online at: https://www.cortecostituzionale.it/actionSchedaPronuncia.do?param_ecli=ECLI:IT:COST:2024:135 (Accessed May 27, 2025).

[23] Italian Constitutional Court, Judgment No. 66 of 2025 (2025). Available online at: https://www.cortecostituzionale.it/actionSchedaPronuncia.do?param_ecli=ECLI:IT:COST:2025:66 (Accessed May 27, 2025).

[24] Fineschi V Turillazzi E Morena D Alesii A. Il suicidio medicalmente assistito alla svolta criteriologica: tre e non più quattro sono i punti fermi decisionali dettati dalla consulta? Responsabilità civile e previdenza. (2024) 89:1728–44.

[25] Bell C Appel CW Pedersen AR Vedsted P. Perceived treatment burden and health-related quality of life in association with healthcare utilization among patients attending multiple outpatient clinics. Health Qual Life Outcomes. (2025) 23:42. doi: 10.1186/s12955-025-02366-y, PMID: 40259350 PMC12013174

[26] Shaffer CS Cook AN Connolly DA. A conceptual framework for thinking about physician-assisted death for persons with a mental disorder. Psychology Public Policy Law. (2016) 22:141–57. doi: 10.1037/law0000082

[27] Trachsel M Hodel MA. Palliative sedation on the grounds of intolerable psychological suffering and its implications for treatment-refractory mental disorders. Bioethica Forum. (2018) 11:45–9. doi: 10.24894/BF.2018.11014

[28] Cassel EJ. The nature of suffering and the goals of medicine. N Engl J Med. (1982) 306:639–45. doi: 10.1056/NEJM198203183061104, PMID: 7057823

[29] Associazione Luca Coscioni. Il caso di Martina Oppelli (2025). Available online at: https://www.associazionelucacoscioni.it/il-caso-di-martina-oppelli (Accessed June 16, 2025).

[30] Stoll J Ryan CJ Trachsel M. Perceived burdensomeness and the wish for hastened death in persons with severe and persistent mental illness. Front Psychiatry. (2021) 11:532817. doi: 10.3389/fpsyt.2020.532817, PMID: 33510652 PMC7835407

[31] Colombo AD Dalla-Zuanna G. Data and trends in assisted suicide and euthanasia, and some related demographic issues. Population Dev Review. (2024) 50:233–57. doi: 10.1111/padr.12605

